# Alterations of the endocannabinoid system in adolescents with non-suicidal self-injury as a function of childhood maltreatment

**DOI:** 10.1038/s41398-024-03205-2

**Published:** 2024-12-18

**Authors:** Marc D. Ferger, Christine Sigrist, Susanne Brodesser, Michael Kaess, Julian Koenig

**Affiliations:** 1 University of Cologne, Faculty of Medicine and University Hospital Cologne, Department of Child and Adolescent Psychiatry, Psychosomatics and Psychotherapy, Cologne, Germany; 2 Department of Child and Adolescent Psychiatry, Centre for Psychosocial Medicine, University of Heidelberg, Heidelberg, Germany; 3 Department of General Psychiatry, Centre for Psychosocial Medicine, Medical Faculty, Heidelberg University, Heidelberg, Germany; 4 University of Cologne, Faculty of Medicine and University Hospital of Cologne, Cluster of Excellence on Cellular Stress Responses in Aging-Associated Diseases (CECAD), Cologne, Germany; 5 University Hospital of Child and Adolescent Psychiatry and Psychotherapy, University of Bern, Bern, Switzerland

**Keywords:** Biomarkers, Physiology, Psychiatric disorders

## Abstract

Non-suicidal self-injury (NSSI) is a highly prevalent phenomenon in adolescence, often associated with prior traumatic experiences. The development and maintenance of NSSI is associated with dysregulation of the stress response, and evidence suggests that the hypothalamic-pituitary-adrenal (HPA) axis plays an important role. The endocannabinoid system is a neuromodulatory system in close functional interaction with the HPA axis. Several studies have reported alterations of the endocannabinoid system in adult patients with post-traumatic stress disorder. However, the role of the endocannabinoid system in children and adolescents with NSSI is less clear, and previously no study examined endocannabinoids in youth with experiences of maltreatment. N-arachidonyl ethanolamide (AEA) and 2-arachidonyl glycerol (2-AG) were quantified alongside sociodemographic and clinical characteristics in *n* = 148 adolescents (12–17 years of age). Analyses addressed group differences comparing healthy controls (HC, *n* = 38), patients with NSSI without (NSSI − CMT, *n* = 42) and with a history of childhood maltreatment (NSSI + CMT, *n* = 68). We show that AEA is reduced in adolescents with NSSI independent of childhood maltreatment. Further, we present first evidence for a negative association between AEA and NSSI frequency as well as AEA and the severity of childhood maltreatment. This is the first study providing evidence for alterations in the endocannabinoid system in children and adolescents engaging in repetitive NSSI. Findings from the study support current endocannabinoid-hypotheses on the neurobiology of trauma and adversity, extending existing findings of altered endocannabinoid signaling following exposure to traumatic events to a well-powered sample of children and adolescents.

## Introduction

Non-suicidal self-injury (NSSI) is defined as the deliberate, self-directed damage of own body tissue without suicidal intent, for purposes not socially or culturally sanctioned [1]. NSSI is a serious clinical problem, particularly in adolescents, as the behavior typically peaks between 14 and 15 years of age and then declines into adulthood [2]. The prevalence of a single episode of NSSI among adolescents is 17.2% in nonclinical samples [3], and girls are more likely to show NSSI[4, 5]. Repetitive NSSI has been observed in several psychiatric conditions [6] most notably depression [7], post-traumatic stress disorder (PTSD) [8], eating- [9] and anxiety disorders [10] and borderline personality disorder (BPD) [11]. NSSI has been proposed as a discrete diagnostic entity in the DSM-5 [12], when the behavior is characterized by five or more episodes of self-harm in the past year. Between 50 and 80% of adolescent psychiatric patients meet this criterion [13]. Importantly, NSSI is a strong predictor of future suicide attempts [14]. NSSI is commonly observed in patients exposed to childhood trauma (i.e., experiences of sexual, physical and emotional abuse as well as physical and emotional neglect during childhood or adolescence) [15–17] and several studies have suggested a potential role of traumatic experiences in its development [18]. However, a substantial group of NSSI patients does not report a history of childhood trauma [15, 16]. Various theoretical models regarding the functions underlying NSSI have been proposed [19, 20]. A meta-analysis has highlighted the importance of intrapersonal functions (emotion-regulation, avoidance of aversive affect, self-punishment) opposed to interpersonal functions (communicating distress, influencing others, seeking support) of self-harm [21]. It is widely accepted that self-harm presents a (dysfunctional) coping strategy to regulate distressing emotional states [21]. Psychotherapy (i.e., dialectical behavior therapy for adolescents (DBT-A) and cognitive behavioral therapy (CBT)), aiming at reducing dysfunctional and promoting functional coping strategies, is effective in the treatment of adolescent NSSI [22]. Neurobiologically informed pharmacological interventions are currently not available for patients with NSSI [22].

A growing body of evidence suggests that biological systems involved in the human stress response contribute to the development and maintenance of NSSI [23]. In brief, alterations in the hypothalamic-pituitary-adrenal (HPA) axis appear to be critical associated with malfunctional stress response and the maintenance of dysfunction coping. Although the exact role of the HPA axis in the neurobiology of NSSI is not yet fully understood, there is considerable evidence that early trauma is an important factor contributing to HPA-axis dysregulation and NSSI [24]. One possible explanation is that chronic exposure to stress and concomitant HPA-axis activation over time [25] leads to habituation, which in turn, results to a hypo-responsiveness to stress [26] that is compensated for by a hyper-responsiveness to pain [27], thus promoting the maintenance of self-harm [28] – such that the absence of normative cortisol secretion following stress can be compensated for by NSSI.

The endocannabinoid system is a neuromodulatory system known to interact with the HPA-axis [29, 30]. The major molecular target of the endocannabinoid receptor is the CB1-receptor, the most highly expressed G protein-coupled receptor in the brain [31, 32]. The CB1-receptor is targeted by two major endogenous ligands, the endocannabinoids N-arachidonyl ethanolamide (anandamide, AEA) and 2-arachidonyl glycerol (2-AG) [33]. While the precise mechanisms underlying the synthesis of AEA and 2-AG are not yet fully understood [34], specific pathways have been identified: for AEA, it is thought to originate from N-acyl-phosphatidylethanolamine (NAPE) through several different pathways [35]. In contrast, the synthesis of 2-AG is hypothesized to involve the sequential activation of phospholipase Cβ and diacylglycerol lipase [36]. The metabolization of these endocannabinoids, however, has been extensively studied and two hydrolytic enzymes, fatty acid amide hydrolase (FAAH) for AEA and monoacylglycerol lipase (MAGL) for 2-AG, have been identified to rapidly degrade the endocannabinoids [37, 38].

In recent years, substantial evidence has emerged suggesting a “gatekeeping” function of tonic endocannabinoid signaling on the HPA-axis [29, 30, 39]. During acute stress, AEA decreases rapidly due to increased FAAH activity to allow for enhanced HPA-axis signaling and thus an effective neuroendocrine response [40]. The latter is thought to be mediated by corticotropin-releasing hormone (CRH)-dependent mechanisms [30, 41, 42], closing the loop between the HPA-axis and the endocannabinoid system. This model is supported by findings of higher corticosterone levels after AEA depletion, an increase in anxiety-like behavior and a deficit in extinction of aversive memory [43]. In addition, after chronic corticosterone exposure FAAH activity increases leading to a reduction in AEA levels [44]. While the vast majority of these findings come from animal studies [45], results from human studies are sparse. In the last years, a growing body of literature has demonstrated alterations in endocannabinoid concentrations in blood [46–48] and hair [49] in patients with PTSD. For a comprehensive review of the endocannabinoid system and PTSD, linking animal models to mechanisms in humans see Ney et al. [50]. Another ligand and namesake of the endocannabinoid system is tetrahydrocannabinol (THC), a psychoactive substance from *Cannabis sativa*. A recent meta-analysis reported an association between cannabis use and an increased prevalence of self-harm [51], further promoting a potential role of the endocannabinoid system in NSSI.

Although several studies have addressed childhood trauma as a key factor in PTSD and a few animal studies investigated the effects of early life adversity on the endocannabinoid system (for an overview see Bassir Nia et al. [52].), previously no study has comprehensively investigated endocannabinoids in children and adolescents with childhood maltreatment. To date, three studies have examined endocannabinoid concentrations in adult patients with BPD: In a pilot study, Wingenfeld et al. found reduced AEA concentrations in the hair of patients with BPD [53] while Schaefer et al. measured higher serum AEA concentrations in patients with BPD [54]. In another recent study [55], Spohrs et al. identified elevated plasma levels of AEA in patients diagnosed with BPD. A key finding from this investigation was the significant influence of the common FAAH genotype (rs324420) on AEA levels. Typically, individuals carrying the A allele of this genotype exhibit higher AEA concentrations compared to those with the CC genotype [56]. Interestingly, this genotype-dependent difference in AEA levels was observed exclusively within the control group and was absent in BPD patients. The absence of relatively decreased AEA levels in C-allele carriers among BPD patients suggests a potential dysregulation of FAAH activity. The authors propose that the observed increase in AEA levels in BPD patients, particularly those with the CC genotype, may serve as a compensatory mechanism and thereby contributing significantly to the overall disparity in AEA levels between patients and controls. Consequently, the interplay between FAAH genotype and AEA regulation appears to be a critical factor in understanding the biochemical underpinnings of the endocannabinoid system in BPD. The aim of the present study was to investigate plasma levels of circulating endocannabinoids and cortisol as a marker of the HPA-axis in adolescent patients with NSSI and to extend the current evidence of altered endocannabinoid signaling and the relationship to the HPA-axis following maltreatment to a large and well-characterized clinical sample of adolescents. Drawing from animal studies examining the interaction between the endocannabinoid system and the HPA axis, we hypothesized an inverse association between AEA and cortisol levels. Based on the existing evidence reviewed, we hypothesized that AEA and 2-AG are reduced in adolescents engaging in NSSI compared to matched healthy controls (HC) and that reduced levels of AEA and 2-AG are associated with the severity of maltreatment history.

## Materials and methods

### Participants

Patients with NSSI were recruited from the specialized outpatient clinic for risk-taking and self-harm behavior “*Ambulanz für Risikoverhalten und Selbstschädigung (AtR!Sk)*” [57] at the Department of Child and Adolescent Psychiatry, University Hospital Heidelberg. After an initial diagnostic assessment, patients were invited within six weeks to participate in the consecutive AtR!Sk-Bio cohort, which aims to identify biological correlates of risk-taking and self-harming behavior in adolescence.

Eligibility criteria for both patients and healthy controls (HC) were age 12–17 years. Patients with NSSI were only included if they reported five or more episodes of NSSI in the past year, according to the diagnostic criteria of the DSM-5 [12]. Patients with acute psychotic symptoms were excluded but since there is a high comorbidity with other psychiatric disorders, other diagnoses were included in the study (see Table 1*)*. Inclusion criteria for HC were no history of NSSI, no endorsement of any psychiatric disorder, and no treatment for any psychiatric disorder (lifetime). Exclusion criteria for both patients and HC were pregnancy, primary neurological, endocrinological, or cardiovascular disease, or lack of speech comprehension.

**Table 1:** Sociodemographic characteristics of the study sample.

Variable		Group; mean ± SD or *N* (%)		*P*^a^
	HC, (*n* = 38)	NSSI-CM, (*n* = 42)	NSSI + CM, (*n* = 68)	
Age (yr)	14.7 ± 1.25	14.8 ± 1.54	14.9 ± 1.49	0.876
Height (cm)	163.9 ± 5.14	165.4 ± 6.81	165.9 ± 6.74	0.339
Weight (kg)	54.42 ± 10.63	61.3 ± 14.23	58.4 ± 12.50	0.059
BMI	20.2 ± 3.34	22.4 ± 4.66	21.2 ± 4.01	0.061
School Type^b^				0.009
Gymnasium	24 (63.2)	17 (40.5)	19 (28.4)	
Realschule	12 (31.6)	14 (33.3)	33 (49.3)	
Hauptschule	1 (2.6)	4 (9.5)	3 (4.5)	
Other	1 (2.6)	7 (16.7)	12 (17.9)	
Cannabis use				0.240
Never	33 (86.8)	34 (81.0)	46 (69.7)	
Regular	4 (10.5)	5 (11.9)	10 (15.2)	
Heavy	1 (2.6)	3 (7.4)	10 (15.2)	
Cortisol (ng/ml)	183.6 (82.63)	167.8 (72.21)	166.7 (64.39)	0.474
NSSI frequency (6 months)	–	31.5 ± 5.51	31.4 ± 4.14	0.996
Age of first NSSI	–	12.6 ± 0.31	12.7 ± 0.20	0.784
Lifetime suicide attempts	–	1.7 ± 0.25	4.5 ± 7.39	0.131
Age at first suicide attempt	–	13.6 ± 0.65	13.5 ± 0.30	0.870
ICD-10 Diagnoses				
F0.X	–	–	–	
F1.X	–	9 (21.4)	15 (22.1)	
F2.X	–	–	–	
F3.X	–	22 (52.4)	44 (64.7)	
F4.X	–	9 (21.4)	35 (51.5)	
F5.X	–	6 (14.3)	9 (13.2)	
F6.X	–	9 (21.4)	29 (42.6)	
F7.X	–	–	–	
F8.X		–	1 (1.5)	
F8.X		10 (23.8)	19 (27.9)	
BPD-Criteria	0.1 ± 0.35	3.0 ± 1.69	3.4 ± 2.22	<0.001
DIKJ	6.0 ± 4.03	25.7 ± 8.68	30.8 ± 8.66	<0.001
SCL-90	0.2 ± 0.17	1.3 ± 0.55	1.7 ± 0.73	<0.001
CECA.Q	–	–	0.5 ± 0.22	

*BMI* body mass index, *BPD* borderline personality disorder, *DIKJ* Depressionsinventar für Kinder und Jugendliche, *SCL-90* Symptom-Checklist-90, *CECA.Q* Childhood Experiences of Care and Abuse questionnaire.

^a^Significance: Values refer to differences between groups, with one-way analysis of variance (ANOVA) for continuous variables in the whole sample, t-tests between the patient groups, and Fisher’s exact test for categorical variables.

^b^Hauptschule: secondary-school terminating with a lower secondary-school level II certificate: Realschule: secondary-school terminating with a secondary-school level I certificate; Gymnasium: secondary-school terminating with the general qualification for university entry.

In addition, based on sex differences in the prevalence of NSSI and sex-specific differences in endocannabinoid signaling [58], only female participants were included in the present analyses. All participants were of Caucasian origin, as heterogeneity in endocannabinoid levels have been observed in ethnically diverse samples [48]. The Ethics Committee of the Faculty of Medicine, University of Heidelberg, approved the scientific evaluation of AtR!Sk (IRB approval number S-449/2013) and the additional neurobiological assessments (IRB approval number S-514/2015). The study was carried out in accordance with the Declaration of Helsinki. All participants and their caregivers provided written informed consent and received an allowance of €40 for their participation.

### General procedures

The study consisted of two separate appointments. The first appointment included diagnostic assessments with the relevant instruments described in detail below. The biological assessment was part of the second appointment, which started at 8 a.m. with measurements of height and weight, as well as questions about participants’ nicotine smoking habits, physical illnesses within the past three months, and regular medication use. To account for potential interference with the blood draw, participants were asked whether they were fasting as instructed, when they had last eaten, and about their cigarette consumption on the day of the assessment. Prior to blood collection, participants were asked to lie in a horizontal position, and a resting ECG was obtained. Fasting blood was drawn from the crook of the arm in this supine position by trained medical personnel. These procedures (including questionnaires, ECG procedure) ensured that all participants had the same level of exercise prior to blood collection for at least 10 min, as exercise prior to blood collection leads to increased AEA concentrations [59].

### Measures

Sociodemographic characteristics were obtained through a semi-structured interview. NSSI and suicide attempts were measured using single items from the German version of the Self-Injurious Thoughts and Behaviors Interview (SITBI-G) [60], a semi-structured interview for the detailed assessment of self-injurious thoughts and behaviors that was slightly modified to meet the DSM-5 criteria for NSSI. The used version was previously evaluated, showing good psychometric properties [60]. BPD symptoms were assessed using the relevant part of the German version of the Structured Clinical Interview for DSM-IV Axis II Personality Disorders (SCID-II), that showed good internal consistency in other studies [61]. Self-reported depressive symptoms were assessed using the Depression Inventory for Children and Adolescents (DIKJ) [62]. The 26 items of the DIKJ were constructed based on the DSM-IV criteria for depression, showing excellent psychometric properties [62]. The Symptom-Checklist-90 (SCL-90), a self-report questionnaire with nine primary symptom dimensions, was used to obtain a comprehensive assessment of psychological symptoms and distress [63]. We used the German translation of the Childhood Experience of Care and Abuse Questionnaire (CECA.Q) [64] to assess childhood maltreatment. The CECA.Q items were taken directly from the interview version and adapted to cover modules for parental care (antipathy and neglect), physical abuse, and sexual abuse. To assess maltreatment severity, a dimensional maltreatment score was created using four modules of the CECA.Q, which showed moderate to excellent internal consistency [65]. Self-reported cannabis use in the past year was assessed at the first appointment in a brief interview with three pre-defined categories (never/regular/heavy).

### Sampling procedures

Venous blood was collected between 8.30 a.m. and 9.00 a.m. Blood-cortisol analysis was performed according to accredited routines at the central laboratories of the Heidelberg University Hospital with immunoassays (ADVIA Centaur® Assay). For endocannabinoid analysis, blood was collected in 2.7 ml EDTA tubes and centrifuged at 2000 *g* for 10 min at 18 °C. Plasma was pipetted into separate aliquots and immediately frozen at −80 °C until analysis. The exact time until centrifugation was recorded, as AEA is subsequently released from blood cells in a time- and temperature-dependent manner [66]. Levels of endocannabinoids in human plasma samples were determined by Liquid Chromatography coupled to Electrospray Ionization Tandem Mass Spectrometry (LC-ESI-MS/MS) using previously described procedures [67, 68]. Endocannabinoid analysis is described in detail in the *Supplementary Material*. Due to rapid isomerization of 2-AG ex vivo during the extraction process and the physiologically negligible amounts of 1-AG, the 2-AG, and 1-AG peaks were integrated and added together to determine the levels of 2-AG, as previously described [46, 69] and the sum of 2-AG/1-AG compounds is referred to as 2-AG throughout the paper.

### Statistical analysis

Prior to analyses, the main variables of interest (NSSI, endocannabinoids and cannabis use) were checked for missing values. AEA and 2-AG concentrations were not normally distributed (indicated by skewness-kurtosis). Therefore, data were log-transformed. Sociodemographic and clinical variables were tested for between-group differences using Fisher’s exact test or χ²-tests and one-way analysis of variance (ANOVA), with group as the between-subjects factor, and Sidak posthoc tests to compare the reference group (HC) with NSSI patients with and without a history of maltreatment respectively. To account for the potential influence of well-known confounders influencing endocannabinoid concentrations, such as age [70], body mass index [71], food intake prior to blood draw [72], time from blood collection to centrifugation [66], and cannabis use [73] were included in the respective regression models, with endocannabinoid AEA or 2-AG as the dependent variable. Analyses of age of NSSI onset, duration of NSSI, the frequency of NSSI episodes and severity of psychiatric symptoms were conducted within the patient group only using two-tailed t-tests to examine the effect of childhood maltreatment. Finally, Pearson product-moment correlations were used to assess associations between NSSI frequency, maltreatment severity and endocannabinoids. All analyses were performed using Stata (Version 17; StataCorp LP, College Station, TX, USA) with a significance level of α = 0.05.

## Results

### Sample characteristics

The final sample for analyses included *n* = 148 adolescents aged 12–17 years (M = 14.82, SD = 0.12). Of these, *n* = 38 (25.68%) were HC, *n* = 42 (28.30%) were patients with NSSI without childhood maltreatment (NSSI-CM) and *n* = 68 (45.95%) were NSSI patients with childhood maltreatment (NSSI + CM). The sociodemographic and clinical characteristics of the groups are shown in Table 1. There were no significant differences in age, BMI, or cortisol. Participants differed significantly on school type, as HC were more likely to attend a *Gymnasium* (secondary-school terminating with the general qualification for university entrance) (Fisher’s exact: χ²_(6)_ = 16.28, *p* = *0.009*). Patients reported incidents of NSSI on a mean of 31.43 (SD = 3.30) days within the past 6 months and there was no significant difference between the NSSI-CM and NSSI + CM groups (*p* = *0.996*). The mean age of onset of NSSI was 12.68 years (SD = 0.17). Patients reported a mean duration of NSSI of 2.17 years (SD = 0.20). There was no difference between the NSSI-CM and NSSI + CM groups in age of NSSI onset and duration of NSSI (*p* = *0.785* and *p* = *.992*). The NSSI + CM included more patients with suicide plans (χ²_(1)_ = 5.41, *p* = *.020*) and suicide gestures (χ²_(1)_ = 4.39, *p* = *0.036*) compared to the NSSI-CM group, but there was no difference in adolescents with suicide attempts (χ²_(1)_ = 0.24, *p* = *0.624*) or the number of lifetime suicide attempts between the NSSI-CM (M = 1.78, SD = 1.06) and the NSSI + CM group (M = 4.48, SD = 7.39, *p* = 0.131). Similarly, NSSI-CM and NSSI + CM groups did not differ in the number of BPD criteria endorsed (*p* = *0.267*). However, the NSSI + CM group scored higher on depressive symptoms (DIKJ) (M = 30.84, SD = 1.06) than the NSSI-CM group (M = 25.68, SD = 1.56, *p* = *0.007*) and showed increased symptom severity (SCL-90) (M = 1.72, SD = 0.10) compared to the NSSI-CM group (M = 1.33, SD = 0.10, *p* = *0.011*).

### Endocannabinoids

**Fig. 1:**
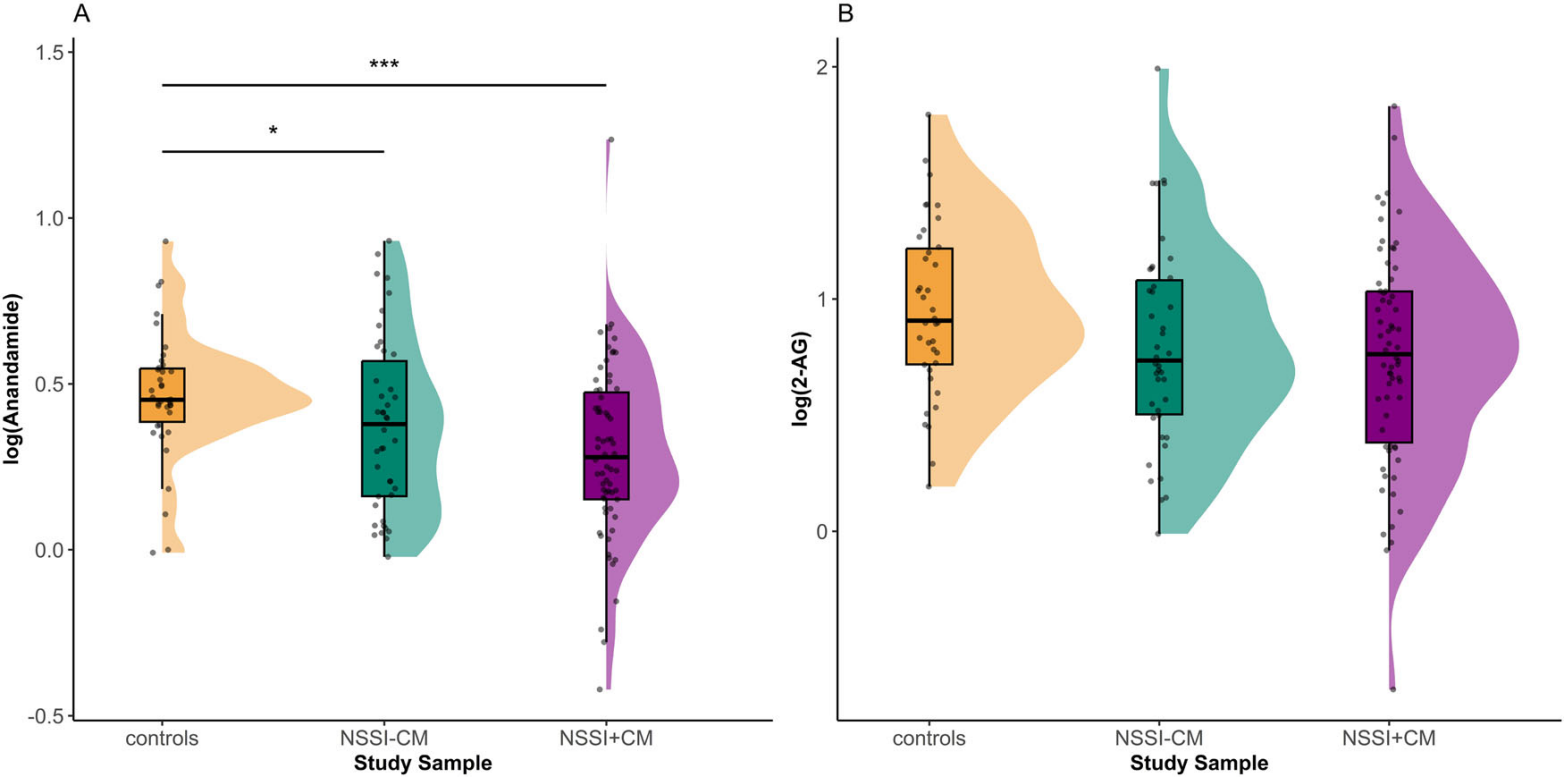
Endocannabinoid concentrations in the study sample. **A** AEA is reduced in both NSSI-CM and NSSI + CM group compared to HC (F_(2, 136)_ = 6.28; *p* < *0.0001*, HC vs. NSSI − CM ß −0.197; *p* = *0.001*, HC vs. NSSI + CM ß −0.266; *p* < *0.0001*). There was no statistical significant difference between the NSSI groups. **B** There were no significant differences in 2-AG levels between the groups (F_(2, 136)_ = 1.99; *p* = *0.061*); *p < 0.05, ***p < 0.001.

**Fig. 2:**
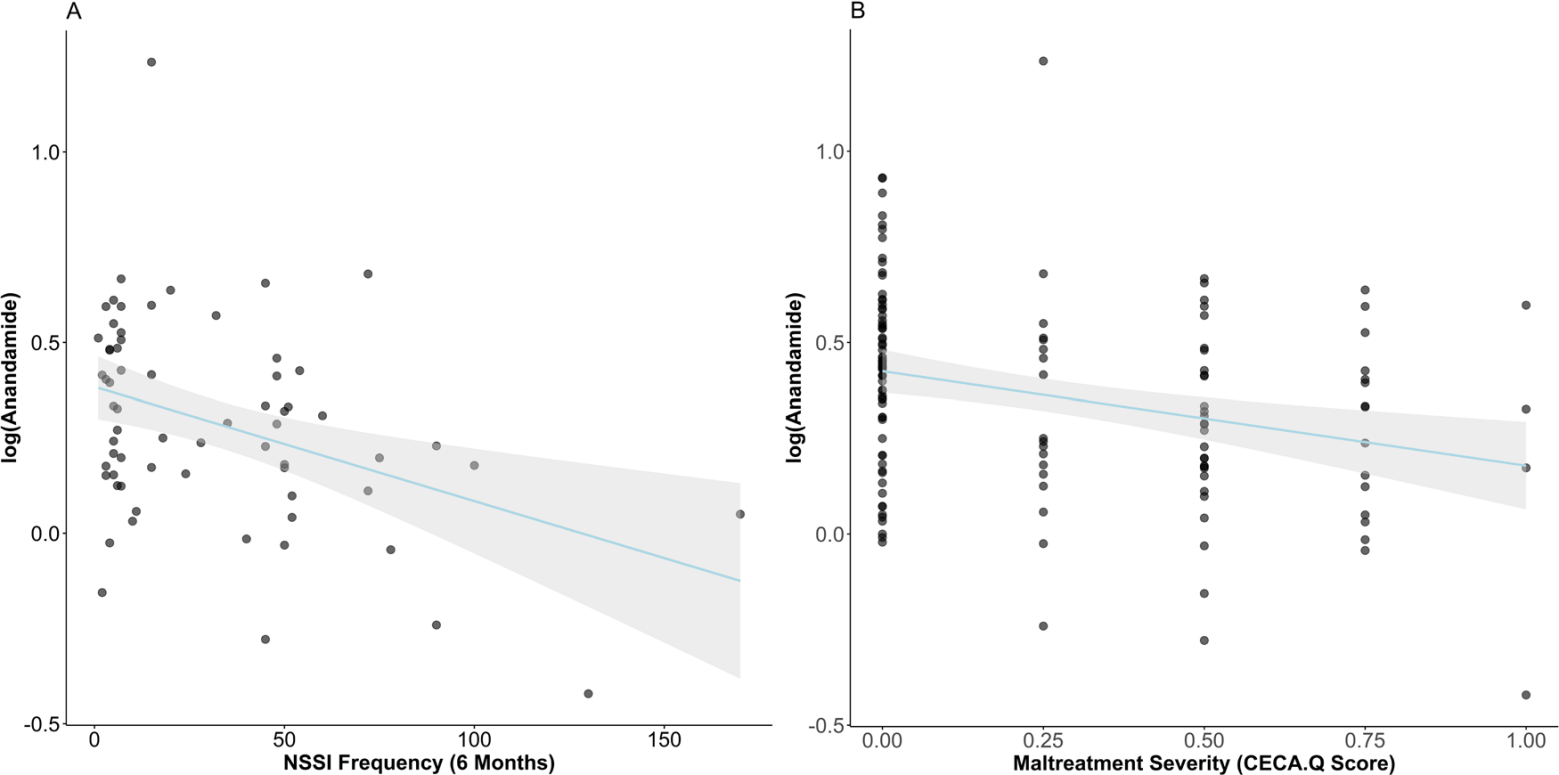
Correlation of anandamide and NSSI frequency and maltreatment severity. **A** AEA was negatively correlated with NSSI frequency in patients with NSSI and maltreatment experience (NSSI + CM) (F_(6, 59)_ = 6.69; *p* < *0.0001*, NSSI frequency: ß = −0.002; *p* = *0.021*) **B** AEA was negatively correlated with maltreatment severity (F_(6, 126)_ = 4.97; *p* < *0.0001*, maltreatment severity: ß = −0.280; *p* < *0.0001*).

In the full sample, we found a significant difference in AEA between groups, with reduced AEA in both NSSI groups compared to HC (F_(2, 136)_ = 6.28; *p* < *0.0001*, HC vs. NSSI − CM ß −0.197; *p* = *0.001*, HC vs. NSSI + CM ß −0.266; *p* < *0.0001*) (Fig. 1A). However, we found no significant differences in 2-AG levels (F_(2, 136)_ = 1.99; *p* = *0.061*) (Fig. 1B). There was no significant association between AEA (*p* = 0.270) or 2-AG (*p* = 0.501) and frequency of self-harm in the full sample NSSI patients. However, AEA was negatively correlated with NSSI frequency in patients with NSSI and maltreatment experience (NSSI + CM) (F_(6, 59)_ = 6.69; *p* < *0.0001*, NSSI frequency: ß = −0.002; *p* = *0.021*) (Fig. 2A). This association was not significant for 2-AG (*p* = *0.385*). There was no association between AEA and the age of NSSI onset (*p* = *0.759*) or the duration of NSSI (*p* = *0.386*). In the full sample, AEA was negatively correlated with maltreatment severity (F_(6, 126)_ = 4.97; *p* < *0.0001*, maltreatment severity: ß = −0.280; *p* < *0.0001*) (Fig. 2B), but there was no significant correlation between 2-AG and maltreatment severity (*p* = *0.087*). In NSSI patients, both AEA (*p* *=* *0.797*) and 2-AG (*p* *=* *0.062*) were not associated with the number of lifetime suicide attempts. The age of first suicide attempt was not associated with current levels of AEA (*p* = 0.067) or 2-AG (*p* = 0.617). Comparing patients with and without a history of suicide attempts, we found no difference in levels of AEA (*p* = 0.313) or 2-AG (*p* = 0.518). Notably, there were no differences in current AEA (*p* = 0.644) or 2-AG (*p* = 0.659) levels between patients who had attempted suicide after making plans and those who had plans but hadn’t attempted suicide yet. There was no association between plasma cortisol levels and either AEA (*p* *=* *0.360*) or 2-AG (*p* = *0.523*) in the whole sample or in the patient group only AEA (*p* = *0.695*) and 2-AG (*p* *=* *0.596*). In the patient groups, there was no significant correlation between AEA (*p* *=* *0.141*) and 2-AG (*p* *=* *0.787*) with the number BPD criteria endorsed, or depression severity (DIKJ) (*p* *=* *0.761* and *p* *=* *0.389*). Both AEA (*p* = *0.154*) and 2-AG (*p* = *0.420*) were not associated with global symptom severity (SCL-90). Finally, we found no association between frequency of cannabis use and endocannabinoid levels (AEA *p* = *0.919*, 2-AG *p* = *0.750*). A full overview on the associations between clinical characteristics and endocannabinoids is available in Supplement Table 1.

### Cannabis use

**Fig. 3:**
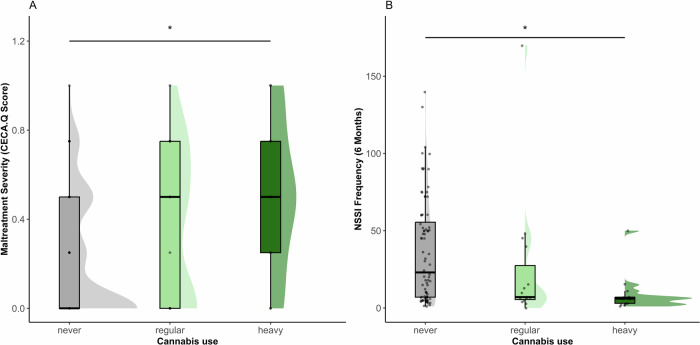
Cannabis use frequency and NSSI frequency and maltreatment severity. **A** Participants with heavy cannabis use reported more severe maltreatment (M = 0.5, SD = 0.34) than those with no cannabis use (M = 0.21, SD = 0.27; F_(2, 132)_ = 7.08; *p* = *0.001*, post hoc: never vs. heavy use *p* = *0.003*) **B** Patients with NSSI who were heavy cannabis users reported fewer episodes of self-harm (M = 9.15, SD = 12.88) compared with those who had not used cannabis in the past year M = 36.34, SD = 33.95, F_(2, 105)_ = 3.95; *p* = *0.023*, post hoc: never vs. heavy use *p* = *0.024*); *p < 0.05.

Among participants, *n* = 113 (77.4%) reported no cannabis use in the past year, *n* = 19 (13.01%) reported regular cannabis use, and *n* = 14 (9.59%) reported heavy cannabis use. There was no significant difference in cannabis use between the three groups (χ²_(4)_ = 5.77, *p* = *0.240*) or between HC and all NSSI patients (Fisher’s exact: χ²_(2)_ = 3.43, *p* = *0.169*). However, adolescents with childhood maltreatment were more likely to use cannabis overall than those without experiences of maltreatment (χ²_(1)_ = 4.08, *p* = *0.043*). In the full sample participants with heavy cannabis use reported more severe maltreatment (M = 0.5, SD = 0.34) than those with no cannabis use (M = 0.21, SD = 0.27; F_(2, 132)_ = 7.08; *p* = *0.001*, post hoc: never vs. heavy use *p* = *0.003*) (Fig. 3A) and endorsed a higher number of BPD criteria (M = 3.71, SD = 2.46) than those with no cannabis use (M = 2.12, SD = 2.11; F_(2, 143)_ = 5.04; *p* = *0.007*, post hoc: never vs. heavy use *p* = *0.034*). Patients with NSSI who were heavy cannabis users reported fewer episodes of self-harm (M = 9.15, SD = 12.88) compared with those who had not used cannabis in the past year (M = 36.34, SD = 33.95, F_(2, 105)_ = 3.95; *p* = *0.022*, post hoc: never vs. heavy use *p* = *0.024*) (Fig. 3B). Patients with NSSI showed no difference in global symptom severity (SCL-90) based on cannabis use (F_(2, 94)_ = 0.29; *p* = *0.737*) and there was no difference in cortisol (F_(2, 143)_ = 0.12; *p* = *0.891*) between the cannabis use groups. Full reporting on differences between groups by cannabis use is available in Supplement Table 2.

## Discussion

The present study is the first to examine the association between endocannabinoids and childhood maltreatment in a clinical sample of adolescents with repetitive NSSI and well-matched typically developing controls. This is the first study to demonstrate alterations in circulating endocannabinoids (reduced AEA) in adolescent patients with NSSI and a negative association between AEA and the frequency of NSSI. Although the endocannabinoid system has been implicated in individuals with trauma for some time, we present the first direct evidence in adolescents with a history of childhood maltreatment. Our finding of a negative association between AEA and maltreatment severity extends the current knowledge from adult populations of altered endocannabinoid signaling after trauma to children and adolescents—suggesting temporal proximity between traumatic events and altered endocannabinoids. However, it remains largely speculative whether reduced AEA precedes the onset of NSSI or whether AEA is reduced as a consequence of repetitive engagement in NSSI.

Consistent with the existing literature on the general association between cannabis use and trauma [74], adolescents with a history of maltreatment reported more frequent cannabis use. While a recent meta-analysis found more frequent cannabis use to be associated with self-harm [51], we found fewer episodes of NSSI among patients reporting heavy cannabis use. This finding supports the notion that cannabis use may serve as a coping strategy in adolescents with NSSI. However, our study design does not allow to draw causal conclusions: we only retrospectively assessed cannabis use in the past year and used a broad self-report questionnaire without further differentiation, which is a limitation in drawing conclusions about underlying mechanisms [75]. Further research on the role of cannabis in NSSI is needed. Consideration of the therapeutic implications of cannabis for patients with NSSI should be based on randomized controlled trials that have yet to be conducted. Furthermore, findings should be interpreted in the light of adolescence as a critical period of ongoing neural development [76, 77]. Cannabis use is associated with altered neurodevelopment, particularly in brain regions with the greatest degree of age-related change [78] and adolescents are more sensitive to the behavioral and cognitive effects of THC [79]. These findings add weight to the argument that the cannabinoid system may be a novel therapeutic target in NSSI and extend the discussion beyond cannabis to the endocannabinoid system.

The findings of reduced AEA and dimensional associations with NSSI frequency are consistent with previous findings of reduced hair AEA in female BPD patients [53]. Similarly, the AEA hydrolyzing enzyme FAAH has been shown to be elevated in the prefrontal cortex of BPD patients [80]. Our data show that there is no association between the duration of NSSI and AEA, suggesting, that the progression of NSSI does not imply a change in AEA concentration over time. If AEA is dysregulated as a result of NSSI, this process is likely to occur relatively early in the course of NSSI or even before NSSI onset. The existing literature only covers longer intervals between trauma exposure and endocannabinoid analysis [46]. The hypothesis of changes in AEA prior to the onset of psychopathology is supported by data from a prospective cohort-study, that showed little within-person variation in AEA but a significant longitudinal between-person relationship between hair AEA and depressive symptoms in adults with depression [81]. Several studies investigating a functional polymorphism of the AEA-degrading enzyme FAAH (C385C- > A), which results in reduced FAAH activity and therefore higher AEA concentrations, have repeatedly shown that carriers with lower FAAH activity exhibit better stress or fear-related behavior [82], e.g., reduced subjective anxiety responses to a stress task [83], improved fear extinction [56] and more successful extinction recall [84]. A recent fMRI study demonstrated a positive association between higher AEA levels and greater neural activation during extinction learning [85] and Zabik et al. showed that FAAH (C385A) is associated with less amygdala activation during extinction recall [86] underscoring the positive behavioral effects of higher AEA from a neuroimaging perspective. The protective effect of higher AEA was further demonstrated by Mayo et al. with C385A carriers showing resistance to stress induced changes in negative affect [56]. Recently, resilience to the development of substance use disorder following childhood trauma was associated with increased levels of AEA at baseline and during a stress task [87]. These findings provide a compelling support for the protective effects of elevated AEA on stress-related disorders. Consistent with this, we show an association between lower AEA and greater frequency of NSSI and lower levels of AEA in patients with NSSI in general. These findings led to the plausible proposal of an *endocannabinoid hypothesis* in the pathophysiology of NSSI suggesting that lower AEA predisposes to engage in NSSI.

In patients who develop NSSI, AEA may be reduced prior to the onset of the behavior. Preclinical studies have demonstrated that the eCB-system regulates the HPA axis [39, 88]. Assuming these findings can be extrapolated to humans, disturbances in the HPA axis due to alterations in the eCB system could be associated with increased emotional reactivity and responsiveness to negative stimuli which in turn leads to increased stress in daily life [89, 90]. To address the impact of childhood trauma from an endocannabinoid-driven hypothesis, we propose that trauma contributes to, but is not necessary for, the development of NSSI: childhood trauma leads to hyperactivation of the HPA axis, and this effect is facilitated by the reduced gatekeeping function of the endocannabinoid system due to lower AEA levels. The absence of differences in AEA levels between NSSI patients with and without a history of childhood maltreatment, may indicate that early adversity itself does not alter endocannabinoid levels and that lower AEA leads to the development of NSSI following early adversity. It seems likely that, in line with the theory of protective effects of higher AEA on the development of stress-related disorders mentioned above, adolescents with lower AEA are more sensitive to the effects of early adversity, as the protective effect of high AEA is absent. Following the idea that NSSI is more common, but not exclusive, in patients exposed to childhood maltreatment, this leads to the suggestion of a ‘double hit’ hypothesis as a result of lower AEA, with higher ‘baseline’ stress in daily life due to higher HPA activity on the one hand, and less protection against stress-induced changes in emotional response on the other. Empirical proofs of this hypothesis remain to be conducted, and in best case more in depth techniques like PET imaging analysis of FAAH activity can shed light onto the underlying mechanisms of the alterations of the eCB-system in patients with NSSI.

Strikingly, the physiological up-regulation of the endocannabinoid system, which has been extensively studied in animals (for an overview see Meyer et al. [76] but has not yet been fully translated to human contexts [91], parallels the prevalence of NSSI, with a peak in early adolescence and a decline toward adulthood. One might speculate that in early childhood slightly reduced AEA is unlikely to lead to NSSI due to compensatory mechanisms. However, in adolescence, when AEA is normatively elevated and sensitive to dysregulation, reduced AEA may exceed the limits of compensatory mechanisms and promote NSSI. As AEA physiologically declines into adulthood, parallel data show less NSSI [2, 92]. However, even though engagement in NSSI may decrease the underlying dysfunctional pattern linking difficulties in stress regulation to reduced AEA as persists, potentially explaining why adolescents who previously engaged in NSSI are more likely to show other stress-related disorders in adulthood.

It is interesting to note that we have not been able to show a direct correlation between endocannabinoids and plasma cortisol. While there is strong evidence from animal studies [30, 93, 94], several human studies with adult participants have also failed to translate these findings of neuroendocrine interaction [56, 95]. One explanation may be the variability of plasma cortisol levels, especially during adolescence, and compensatory mechanisms of the HPA axis, such as a corresponding down-regulation as proposed in the “attenuation hypothesis” [26]. For example, we have recently shown that NSSI patients do not show a gradual increase in pituitary volume with age compared to healthy controls, which may indicate altered pituitary maturation in NSSI [96]. In addition, a recent meta-analysis of various markers of HPA-axis function in child maltreatment found effects only on cortisol stress reactivity but not on basal cortisol levels [97]. The interplay between the HPA axis and the endocannabinoid system in humans remains complex, and more translational research is needed. Further research should focus on the investigation of endocannabinoids and cortisol stress reactivity in patients who engage in NSSI.

In light of the present findings, FAAH inhibitors, which have been shown to increase AEA levels [95], may represent a promising new class of drugs in the treatment of NSSI. We suggest that adolescent patients with low levels of AEA may benefit from such pharmacological enhancement, as higher levels of AEA protect against stress-induced changes in the development of corticolimbic structures, which are known to occur in NSSI [96]. Furthermore, exercise is known to increase AEA [59] and may be a non-pharmacological strategy that has recently been shown to enhance the beneficial effects of psychotherapy in PTSD[98]. However, the magnitude and lasting duration of exercise-induced increases in AEA are inferior compared to pharmacological enhancement with FAAH inhibitors.

### Limitations and outlook

One significant limitation of our study lies in our sole reliance on self-reported cannabis use data with no standardized questionnaire from the past year, without access to recent cannabis use confirmed by an objective measure such as urine toxicology. It is well-established that cannabis use influences the endocannabinoid system [99] and recent findings have elucidated a correlation between cannabis use and reduced levels of anandamide in adult patients with psychosis [100]. In a randomized, double-blind study with healthy volunteers, Chester et al. investigated the effects of cannabis on the human endocannabinoid system, revealing elevated plasma AEA levels shortly after inhalation, followed by a decrease in AEA levels with repeated sessions of cannabis use [73].

In our analysis, we controlled for cannabis use within the past year and found consistent results regarding endocannabinoid findings. Given that Chester et al.‘s randomized controlled trial demonstrated no significant alterations in plasma endocannabinoid levels beyond 10 minutes post-cannabis inhalation and considering our study’s design involved direct supervision for longer durations prior to the blood draw, it is unlikely that very recent cannabis use significantly influenced endocannabinoid levels. Nonetheless, future investigations should explore cannabis use with greater granularity and incorporate objective markers such as urine toxicology for more comprehensive assessment. It is pertinent to note as a limitation that while the prevalence of substance use disorder is comparatively low among children and adolescents in contrast to adults, future studies should still prioritize controlling for other drugs, particularly alcohol and opioids. This precaution is warranted due to their recognized impact on the endocannabinoid system [101, 102]. Another limitation of our study is the preanalytical processing, particularly the centrifugation of blood samples at room temperature (18 °C) [103]. It has been reported that AEA concentrations in whole blood can increase approximately threefold at room temperature and twofold when samples are kept on ice [104]. Future studies should therefore exercise caution and adhere to stringent protocols during preanalytical processing to minimize variability and ensure the accuracy of AEA measurements. Additionally, in light of the differential responses within the endocannabinoid system to childhood versus adult trauma, primarily informed by insights from animal models [105], an investigation into the repercussions of trauma experienced prior to or during adolescence on the endocannabinoid system emerges as a crucial pathway for future translational research initiatives.

In our investigation, we did not observe any statistically significant correlation between endocannabinoid levels and suicide attempts. It is crucial to highlight that all participants in this study had access to treatment, as it was conducted within the framework of our specialized outpatient clinic for children and adolescents exhibiting NSSI. Therefore, the findings concerning suicide attempts and the endocannabinoid system should be construed solely within the context of a sample seeking assistance and receiving treatment. Given that our research included only female adolescents, extending investigations to encompass all genders is crucial for a more complete understanding of the endocannabinoid system in patients with NSSI.

## Conclusion

This is the first study to demonstrate altered endocannabinoid levels in adolescent patients with NSSI and childhood maltreatment. In line with previous research in PTSD we propose that AEA is a key factor in the development and/or maintenance of trauma-related disorders, including NSSI. We suggest that pharmacologically increasing AEA, for example by FAAH-inhibition may be a novel treatment option for patients with or at risk for developing NSSI.

## Supplementary information

Supplemental Material

## Data Availability

Due to the nature of this research project, participants did not provide consent for their data to be shared publicly, so supporting data is not publicly available. However, anonymized data can be made available upon request from the corresponding author.
