# Supplementary material for: Alterations of the endocannabinoid system in adolescents with non-suicidal self-injury as a function of childhood maltreatment
Source: Transl Psychiatry. 2024 Dec 18;14:491. doi: 10.1038/s41398-024-03205-2 (PMC11655887; doi:10.1038/s41398-024-03205-2)
Supplement: Supplementary file 1 — Supplemental Material [file 41398_2024_3205_MOESM1_ESM.docx]

**Supplemental Material**

*Endocannabinoid Analyses:*

Extraction of plasma samples was performed as previously described (Zoerner et al. 2012) with several modifications: After thawing on ice, 400 µl of plasma were diluted with 600 µl of ice-cold PBS. To all samples 10 µl of an internal standard mixture, containing 100 pg/µl of each of the deuterated endocannabinoids 2-arachidonoyl(20:4) glycerol-d11 and arachidonoyl(20:4) ethanolamide-d4 (Cayman Chemical) were added. After incubation on ice for 15 minutes, 1 ml of toluene was added. Extraction was performed by shaking the samples in a ThermoMixer (Eppendorf) for 5 min at 4 °C and 2000 rpm. After centrifugation (16,100 RCF, 5 min, 4 °C), the upper (organic) phase was transferred to a new tube and the lower phase extracted with additional 800 µl of toluene. The combined organic phases were dried under a stream of nitrogen. The dried extract was resolved in 300 µl of methanol/water 3:1 (v/v). The samples were again centrifuged, and 70 µl of supernatant were transferred to autoinjector vials.

LC-MS/MS analysis was performed by injection of 35 µl of sample onto a Acquity BEH Shield RP18 column (100 mm × 2.1 mm ID, 1.7 µm particle size, Waters) and detection using a QTRAP 6500 triple quadrupole/linear ion trap mass spectrometer (SCIEX). The LC (Nexera X2 UHPLC System, Shimadzu) was operated at 60 °C and at a flow rate of 0.5 ml/min with a mobile phase of 2 mM ammonium acetate in water (solvent A) and 2 mM ammonium acetate in methanol (solvent B). Endocannabinoids were eluted with the following gradient: initial, 75 % B; 0.50 min, 75 % B; 5.00 min, 79 % B; 5.50 min, 90 % B; 5.60 min, 75 % B; 7.50 min, 75 % B (Zoerner et al. 2012).

Endocannabinoids were monitored in the positive ion mode with their specific Multiple Reaction Monitoring (MRM) transitions (Zoerner et al. 2012; Williams et al. 2007). The instrument settings for nebulizer gas (Gas 1), turbogas (Gas 2), curtain gas, and collision gas were 70 psi, 55 psi, 40 psi, and medium, respectively. The Turbo V ESI source temperature was 600 °C, and the ionspray voltage was 5 kV. For all MRM transitions the values for the entrance potential and the cell exit potential were 10 V and 8 V, respectively. The collision energies ranged from 17 to 30 V, the declustering potentials from 70 to 110 V.

The LC chromatogram peaks of endogenous endocannabinoids and deuterated internal standards were integrated using the MultiQuant 3.0.2 software (SCIEX). Endogenous endocannabinoids were quantified on the basis of external calibration curves which were calculated from LC-MS/MS measurements of serially diluted synthetic 2-arachidonoyl glycerol and arachidonoyl ethanolamide standard solutions within the range of 0.0 to 42.3 pmol on column. To each dilution fixed amounts of the deuterated internal standards were added. The standard calibration curves were plotted based on molar concentration versus peak area ratio of endocannabinoid standards to deuterated internal standards. Linearity and correlation coefficients (R2) of the calibration curves were obtained via linear regression analysis. R2 of the calibration curves were >0.99. The calculated amounts of endogenous ethanolamides were normalized to the volume of the plasma sample.

**Supplemental Tables**

Supplemental Table 1. Associations between clinical characteristics and endocannabinoids
(NSSI patients *n*=110)

| Variable |  | **AEA** | | **2-AG** |
| --- | --- | --- | --- | --- |
|  | ß | *p* | ß | *p* |
| Episodes of Self-Harm (6 months) | -0.001 | 0.270 | -0.001 | 0.501 |
| Age of first Self-Harm | 0.004 | 0.759 | 0.014 | 0.568 |
| Duration of Self-Harm | -0.004 | 0.739 | -0.016 | 0.483 |
| BPD-Criteria | -0.017 | 0.141 | 0.006 | 0.787 |
| DIKJ | -0.001 | 0.761 | -0.004 | 0.389 |
| SCL-90 | -0.052 | 0.154 | -0.054 | 0.420 |
| CECA.Q | -0.139 | 0.112 | -0.133 | 0.407 |
| Cortisol | 0.001 | 0.695 | 0.001 | 0.596 |

Supplemental Table 2. Characteristics of the study sample by cannabis use in the past year

| Variable |  | Group; mean ± SD or *N* (%) | | *P*^1^ |  |
| --- | --- | --- | --- | --- | --- |
|  | never, (n=113) | regular, (n=19) | heavy, (n=14) |  |  |
| Age (yr) | 14.6±1.46 | 15.4±1.17 | 15.4±1.34 | 0.023 |  |
| Height (cm) | 164.9±6.73 | 166.4±6.14 | 166.2±3.83 | 0.547 |  |
| Weight (kg) | 58.0±12.48 | 58.9±16.46 | 60.1±9.97 | 0.829 |  |
| BMI | 21.3±4.05 | 21.2±5.09 | 21.7±3.27 | 0.916 |  |
| School Type^2^ |  |  |  | 0.496 |  |
| Gymnasium | 48 (42.5) | 9 (47.37) | 3 (21.43) |  |  |
| Realschule | 44 (38.9) | 6 (31.58) | 8 (57.14) |  |  |
| Hauptschule | 6 (5.31) | 2 (10.53) | 0 (0.00) |  |  |
| Other | 15 (13.27) | 2 (10.53) | 3 (21.43) |  |  |
| Episodes of Self-Harm (6 months) | 36.3±33.95 | 25.3±43.07 | 9.2±12.88 | 0.022 |  |
| Age of first Self-Harm | 12.7±1.90 | 13.1±1.06 | 12.4±1.89 | 0.523 |  |
| BPD-Criteria | 2.1±2.11 | 3.3±2.47 | 3.7±2.46 | 0.007 |  |
| DIKJ | 21.7±13.35 | 25.2±12.21 | 25.8±11.14 | 0.385 |  |
| SCL-90 | 1.2±0.87 | 1.3±0.89 | 1.3±0.83 | 0.737 |  |
| CECA.Q | 0.2±0.27 | 0.4±0.35 | 0.5±0.34 | 0.001 |  |
| BMI = body mass index; BPD= Borderline Personality Disorder; DIKJ = *Depressionsinventar für Kinder und Jugendliche*; SCL-90 = Symptom-Checklist-90 ; CECA.Q =  *Childhood Experiences of Care and Abuse* questionnaire.  ^1^ Significance: Values refer to differences between groups, with one-way analysis of variance (ANOVA) for continuous variables in the whole sample, t-tests between the patient groups and Fisher’s exact test for categorical variables.  ^2^ Hauptschule: secondary school terminating with a lower secondary-school level II certificate: Realschule: secondary school terminating with a secondary-school level I certificate; Gymnasium: secondary school terminating with the general qualification for university entry. | | | | | |
